# [Retracted] Long non-coding RNA ANRIL knockdown suppresses apoptosis and pro-inflammatory cytokines while enhancing neurite outgrowth via binding microRNA-125a in a cellular model of Alzheimer’s disease

**DOI:** 10.3892/mmr.2025.13772

**Published:** 2025-12-08

**Authors:** Bingling Zhou, Lijuan Li, Xin Qiu, Jiashun Wu, Lei Xu, Wei Shao

Mol Med Rep 22: 1489–1497, 2020; DOI: 10.3892/mmr.2020.11203

Following the publication of the above paper, it was drawn to the Editor's attention by a concerned reader that certain of the cell apoptotic data shown in Fig. 5A were strikingly similar to data appearing in different form in another article written by different authors at different research institutes that had already been published in the journal *Cell Cycle;* moreover, the lens smudging patterns underlying the neurite outgrowth experimental data shown in Figs. 2D and 5C matched that of data shown in other figures of the same article published in journal *Cell Cycle*, suggesting these data may have been derived from the same original source.

Owing to the fact that the contentious data mentioned above had already apparently been published previously, the Editor of *Molecular Medicine Reports* has decided that this paper should be retracted from the Journal. The authors were asked for an explanation to account for these concerns, but the Editorial Office did not receive a reply. The Editor apologizes to the readership for any inconvenience caused.

